# Genotype harmonizer: automatic strand alignment and format conversion for genotype data integration

**DOI:** 10.1186/1756-0500-7-901

**Published:** 2014-12-11

**Authors:** Patrick Deelen, Marc Jan Bonder, K Joeri van der Velde, Harm-Jan Westra, Erwin Winder, Dennis Hendriksen, Lude Franke, Morris A Swertz

**Affiliations:** University of Groningen, University Medical Center Groningen, Genomics Coordination Center, Groningen, the Netherlands; Department of Genetics, University of Groningen, University Medical Center Groningen, Groningen, the Netherlands

**Keywords:** GWAS, Imputation, Meta-analysis, Linkage disequilibrium

## Abstract

**Background:**

To gain statistical power or to allow fine mapping, researchers typically want to pool data before meta-analyses or genotype imputation. However, the necessary harmonization of genetic datasets is currently error-prone because of many different file formats and lack of clarity about which genomic strand is used as reference.

**Findings:**

Genotype Harmonizer (GH) is a command-line tool to harmonize genetic datasets by automatically solving issues concerning genomic strand and file format. GH solves the unknown strand issue by aligning ambiguous A/T and G/C SNPs to a specified reference, using linkage disequilibrium patterns without prior knowledge of the used strands. GH supports many common GWAS/NGS genotype formats including PLINK, binary PLINK, VCF, SHAPEIT2 & Oxford GEN. GH is implemented in Java and a large part of the functionality can also be used as Java ‘Genotype-IO’ API. All software is open source under license LGPLv3 and available from http://www.molgenis.org/systemsgenetics.

**Conclusions:**

GH can be used to harmonize genetic datasets across different file formats and can be easily integrated as a step in routine meta-analysis and imputation pipelines.

## Background

Genome-wide association studies (GWAS) increasingly require the integration of multiple genetic data sets to reach sufficient resolution and statistical power, either by imputing missing genotypes or by pooling datasets for a meta-analysis. However, there are two major challenges to be resolved: 1) the large number of different file formats used by the genetics community, and 2) the ambiguous A/T and G/C single nucleotide polymorphisms (SNPs) for which the strand is not obvious. For many statistical analyses, such as meta-analyses of GWAS [1] and genotype imputation [2], it is vital that the datasets to be used are aligned to the same genomic strand.

Genotype data can be coded on either the forward genomic strand or the reverse genomic strand (e.g. a SNP coded T/G on the forward strand would be coded A/C on the reverse strand). The strand used to store the genotypes is not always the same within a dataset (i.e. the same strand may not be used for all variants) or between the different datasets to be aligned (i.e. the same strand may not be used for a variant present in both datasets); these differences can be intentional [3] or accidental. To complicate matters, most of the common file formats do not define the strand used. For some types of SNPs, it is fairly straightforward to detect and correct the strand differences. For example, a T/G SNP is non-ambiguous as its complement on the other strand is A/C. However, G/C and T/A variants are ambiguous or cryptic as their complementary alleles are C/G and A/T, respectively. This ambiguity means it is more difficult to detect and resolve strand issues for these SNPs.

Of course, it is possible to simply exclude all ambiguous variants, however, modern genotyping chips often contain many A/T and G/C SNPs; the ImmunoChip has 25,740 such SNPs (1.7% of all SNPs), the ExomeChip 244,771 (11.9%) and the Omni5-quad 144.578 (3.4%). Simply excluding these variants will limit the power of a GWAS meta-analysis where the A/T or G/C variant is the causal variant or is in higher LD to the causal variant. In the case of imputation it has also been shown that more input genotypes yield imputed genotypes of higher quality [4], so if it is possible to include the A/T and G/C variants, this is more desirable. In the cases where the strand of the genotypes is known, there are many solutions to easily correct the strands of one dataset or to simply state explicitly the strand used, for example as is possible in IMPUTE2 [5] or METAL [6]. In practice, however, this information is not always available or trustworthy.

One solution to the problem of unknown strands is to compare the minor allele between two datasets. However, use of the minor allele is not ideal as it can differ between datasets and populations, especially for common variants. PLINK [7] employs a more powerful approach to detect strand inconsistencies between cases and controls. However, this method requires many manual steps, re-coding of phenotypes before and after the actual alignment, manual alignment of the non-ambiguous SNPs and merging the data into one dataset, and finally a script needs to be written to parse the alignment results from PLINK to determine the actual alignment. When using PLINK, it is not possible to align genotypes with posterior probabilities.

### Implementation

Here, we present Genotype Harmonizer (GH): a new command-line tool to automate genotype data harmonization. GH can read commonly used file formats (PLINK, binary PLINK, VCF, SHAPEIT2 & Oxford GEN) and align a study dataset to a specified reference without any prior knowledge of the strand used. After alignment, GH writes data back to a chosen format (PLINK, binary PLINK, SHAPEIT2 or Oxford GEN). All handling of the genotype data and loading genotypes from the different formats is implemented in our Genotype IO library, which also allows integration of the harmonization tools into other software. GH consists of 25,000 lines of code with a high unit test coverage of over 60% at conditional level and continuous build testing. GH is written in Java and has been tested under Linux, Windows, and OS-X. All source code is available at http://www.github.com/molgenis/systemsgenetics.

GH implements a fully automated method that assigns the strand of ambiguous SNPs by selecting nearby non-ambiguous SNPs that are in linkage disequilibrium (LD) in both the study data and the reference data. GH correlates the estimated haplotype frequencies between the study data and the reference data. If GH finds more negative correlations than positive ones in haplotype frequencies, the ambiguous SNP is swapped to the other strand. When GH is unable to align a SNP (e.g. because of a lack of surrounding SNPs), this ambiguous SNP is excluded from the set. It is possible to prevent exclusion of variants that could not be aligned using LD, GH can optionally perform alignment using the minor allele for variants that have a minor allele frequency below a specified value.

## Findings

### Usage in an imputation workflow

**Figure 1:**
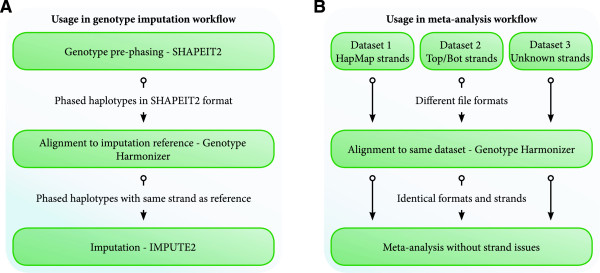
**Usage of Genotype Harmonizer. A)** GH can be applied after the pre-phasing of the genotypes, preventing the need to redo the phasing for each new version of a haplotype reference set. **B)** GH can be used to align and reformat genotype datasets allowing easy merging or meta-analysing of data. By aligning all datasets to a public reference, the genotype data can be kept private by consortia members.

We advise applying GH to pre-phased data before imputation. When pre-phasing using SHAPEIT2 [8] and imputing using IMPUTE2, GH can read the SHAPEIT2 output directly and can write aligned results in the same format for direct use by IMPUTE2 (Figure 1). Performing the alignment after the pre-phasing step ensures that pre-phasing does not need to be repeated when imputing using a different reference set or a newer version of a reference set. GH can also update the variant identifiers of the study data to match the reference set identifiers using the --update-id option. An example command is:

GenotypeHarmonizer.sh --input shapeit2Output --ref

refInVcf --output targetPath --update-id

### Usage to harmonize GWAS data

GH can also be used in merging or meta-analysis of different GWAS datasets (Figure 1). One of the datasets can be used as a reference and the other datasets can be aligned to it, or all the cohorts can be aligned to a public reference set. It is possible to include all the variants present in the study data that are not in the reference set using the --keep option. After alignment the datasets can be investigated using a meta-analysis or can be merged into a single dataset. An example command is:

GenotypeHarmonizer.sh --input dataset1 --ref

dataset2 --output dataset1Aligned --update-id --keep

### Performance

GH requires 6:35 minutes to align a GWAS dataset consisting of 168,408 SNPs and 25,169 samples in binary PLINK format to another GWAS dataset with 528,969 SNPs and 11,950 samples, using a Linux system, a single core and 4 GB of RAM. Aligning the SHAPEIT2 results (25,169 and 19,321 variants on chromosome 1) to the Genome of The Netherlands imputation reference (499 samples, 1,536,126 SNPs on chromosome 1) [9] took 36 seconds using a single core and <1 GB of RAM.

### Comparison using PLINK alignment

We compared the alignment of ambiguous variants using GH to the alignment using the flip-scan option in PLINK. We performed this analysis by using the latest HapMap3 data. We randomly assigned the samples into two equally sized sets, henceforth denoted as set1 and set2. In set1 we randomly changed the strand of roughly 50% of the A/T and G/C variants.

Set1 was aligned using GH by using set2 as the reference using the default settings. We successful aligned 40,617 out of the 55,517 swapped variants, 14 (0.03%) variants were aligned to the incorrect strand. In total 29,801 A/T and G/C variants (27% of the total ambiguous variants) were excluded since there were not enough variants in LD for accurate alignment. There were no variants swapped by GH that were not flipped in our test set.

For the analysis using PLINK we denoted the samples in set1 as cases and set2 as controls; we merged both sets and used the flip-scan option using the default settings. PLINK does not actually report which variants should be swapped but instead provides a log with information on which the decision to swap a variant can be based. Since the PLINK manual does not provide a recommendation on how to select the variants to swap based on this file, we used the same criteria as those used by the GH, i.e. there need to be at least 3 variants in LD, and then we assessed if there were more positive than negative correlations. This resulted in the successful alignment of 37,402 SNPs and the incorrect alignment of 54 SNPs (0.14%); 36,390 (33% of the total ambiguous variants) variants were excluded because of lack of variants in LD. We thus find that the number of incorrectly aligned SNPs increased by 40 SNPs and the number of excluded SNPs increased by 22% from 29,801 to 36,390 when using PLINK instead of GH.

Moreover, in one command GH covers many separate steps which require considerable manual work or scripting when using PLINK: manual alignment of non-ambiguous variants (which PLINK cannot do automatically), conversion of reference haplotypes to a PLINK supported format, merging the reference and study datasets, recoding using a fake phenotype file, running PLINK flip-scan to find swapped SNPs, and the selection and swapping of the SNPs on the wrong strand.

## Conclusions

We have shown that using Genotype Harmonizer we can provide near perfect alignment of ambiguous SNPs without any prior knowledge of the strands. Compared to PLINK we have improved the strand alignment and limited the number of manual steps without sacrificing run-time performance. Another advantage of GH over PLINK is our support of file formats storing haplotype phase or genotype probability information, which also makes our software useful to employ within an imputation workflow or on data that has already been imputed.

GH uses an advanced LD-based method to perform the alignment of ambiguous SNPs and supports many genotype file formats. The underlying Genotype IO API is part of the MOLGENIS open source suite [10], which is also used by several other genetic analysis tools, and we expect the number of supported formats to grow in the future. These enhancements will be made available in later releases of GH. We have used GH to harmonize over 15 imputations and GWAS datasets [11–14]. GH is now a standard part of our imputations and has been applied to over 25,000 samples (publications in preparation). We expect GH to be a major time saver for many research groups and to become a standard part of many analysis pipelines, as it alleviates manual steps when imputing data or when working with multiple GWAS datasets.

## Availability and requirements

**Project name:** Genotype Harmonizer

**Project home page:**http://www.molgenis.org/systemsgenetics

**Operating system(s):** Platform independent

**Programming language:** Java

**Other requirements:** Java 1.6 or higher

**License:** LGPLv3

**Any restrictions to use by non-academics:** Free to use

## Abbreviations

GH: Genotype harmonizer
GWAS: Genome-wide association study
SNP: Single nucleotide polymorphism
LD: Linkage disequilibrium.
